# Metal-Based Nanoparticles with Biostimulatory Effects: Harnessing Nanotechnology for Enhanced Agricultural Sustainability

**DOI:** 10.3390/ma18133142

**Published:** 2025-07-02

**Authors:** Valentina Anuta, Alexandru Blidaru, Cristina-Elena Dinu-Pîrvu, Radu Claudiu Fierascu, Irina Fierascu, Daniela-Ionela Toma (Sărdărescu), Lacramioara Popa, Mihaela Violeta Ghica, Razvan-Mihai Prisada

**Affiliations:** 1 Department of Physical and Colloidal Chemistry, Faculty of Pharmacy, “Carol Davila” University of Medicine and Pharmacy, 6 Traian Vuia Street, 020956 Bucharest, Romania; valentina.anuta@umfcd.ro (V.A.); lacramioara.popa@umfcd.ro (L.P.); mihaela.ghica@umfcd.ro (M.V.G.); razvan.prisada@umfcd.ro (R.-M.P.); 2 Innovative Therapeutic Structures Research and Development Centre (InnoTher), “Carol Davila” University of Medicine and Pharmacy, 6 Traian Vuia Str., 020956 Bucharest, Romania; 3 Surgical Oncology Department, “Prof. Dr. Alexandru Trestioreanu” Oncological Institute, “Carol Davila” University of Medicine and Pharmacy, 25232 Fundeni Street, 022328 Bucharest, Romania; alexandru.blidaru@umfcd.ro; 4 National Institute for Research & Development in Chemistry and Petrochemistry—ICECHIM Bucharest, 202 Spl. Independentei, 060021 Bucharest, Romania; irina.fierascu@icechim.ro; 5 Faculty of Chemical Engineering and Biotechnology, National University of Science and Technology Politehnica Bucharest, 1-7 Gheorghe Polizu St., 011061 Bucharest, Romania; ionela.toma93@yahoo.com; 6 Faculty of Horticulture, University of Agronomic Sciences and Veterinary Medicine of Bucharest, 59 Marasti Blvd, 011464 Bucharest, Romania; 7 National Research and Development Institute for Biotechnology in Horticulture—INCDBH, 37 Bucuresti-Pitesti Str., 117715 Ștefănești, Romania

**Keywords:** metal nanoparticles, sustainable agriculture, biostimulants, plant growth promoter, environmental stress

## Abstract

The application of nanoparticles in agriculture has garnered significant attention due to their potential to enhance plant growth, resistance to stress, and overall productivity. Nanoparticles can trigger physiological and biochemical changes in plants, promoting growth under both optimal and suboptimal environmental conditions. This review explores the mechanisms by which nanoparticles interact with plants, focusing on their role in improving nutrient uptake, stimulating growth, enhancing stress tolerance, and modulating plant metabolic pathways. Furthermore, it examines metal-based nanoparticles that have shown promising biostimulatory effects, their synthesis methods, and their applications in different agricultural systems. Despite the promising results, challenges remain, such as toxicity, environmental impact, and regulatory hurdles, which are crucial for the safe integration of nanoparticles into agricultural practices. The present review article aims to provide a brief overview of the current state of research on nanoparticle-based plant growth enhancers, and their potential to revolutionize sustainable agriculture.

## 1. Introduction

The global population is projected to reach nearly 10 billion by 2050, which presents significant challenges for food production, environmental sustainability, and natural resource management [1]. To meet the increasing food demand while maintaining ecological balance, agricultural practices must evolve to become more efficient, productive, and sustainable. Traditional methods of improving crop yields, such as the extensive use of synthetic fertilizers, pesticides, and herbicides, have led to adverse environmental effects, including soil degradation, water pollution, and the loss of biodiversity [2,3]. Therefore, there is a growing need to explore alternative approaches that can optimize agricultural productivity while minimizing environmental harm. One promising solution lies in the use of plant biostimulants.

Plant biostimulants are natural or synthetic substances that, when applied to plants, stimulate growth, enhance nutrient uptake, and improve resistance to abiotic and biotic stress, and increase overall plant health without being classified as traditional fertilizers or pesticides [4]. Unlike conventional agrochemicals, which directly alter nutrient availability or control pests, biostimulants work by enhancing the physiological and biochemical processes in plants [5]. They are not intended to provide nutrients in large quantities, but instead to optimize plant growth conditions, thus enabling plants to grow more efficiently and withstand various environmental stressors, such as drought, salinity, and temperature extremes [6].

The definition of plant biostimulants, as outlined by the European Union Regulation (2019), encompasses a wide range of substances, including humic and fulvic acids, seaweed extracts, protein hydrolysates, amino acids, and microorganisms, among others [7]. These substances are applied to crops to trigger a series of plant responses that promote growth and yield. Importantly, biostimulants are seen as a tool to reduce the dependency on synthetic chemicals, thus supporting more sustainable agricultural practices [8]. The literature data and different regulatory bodies provides different definitions for this type of materials, as presented in a recent work [7]. A more comprehensive definition was recently provided by Jiang et al. [9], who considered biostimulants to include any class of compounds (including, but not limited to, humic-based, protein-based, oligosaccharide-based, or metabolites-based materials, inorganic substances, or microbial inoculants) which were proven to “help plants with growth and defense” [9].

Biostimulants act through different mechanisms, including the enhancement of nutrient availability, improvement of root development, stimulation of the plant’s defense systems, modulation of hormonal balance, and the promotion of beneficial microbial activity in the rhizosphere [10]. Moreover, plant biostimulants are believed to offer a potential solution for enhancing crop performance in both conventional and organic farming systems, with the ability to boost productivity in a more environmentally friendly manner [11].

In recent years, plant biostimulants have attracted significant interest as part of the movement toward sustainable agriculture. However, there are still some challenges related to understanding their modes of action, determining optimal application methods, and ensuring regulatory approval for their widespread use.

Nanotechnology has emerged as a groundbreaking field with vast applications across various industries, including agriculture. In the agricultural sector, nanotechnology offers innovative solutions that address the inefficiencies and limitations of traditional agricultural practices [12]. The use of nanomaterials, such as nanoparticles (NPs), can revolutionize plant nutrition, pest control, and overall crop management), due to their unique properties, including high surface area, reactivity, and ease of functionalization. Nanotechnology enables the development of more precise, efficient, and environmentally friendly agricultural inputs, helping farmers achieve higher yields with fewer inputs [13].

One of the most significant advantages of nanotechnology in agriculture is its ability to improve the delivery of nutrients and agrochemicals to plants. Conventional fertilizers often suffer from poor efficiency due to their low bioavailability, runoff, and leaching. In contrast, nanoparticles can be engineered to deliver nutrients in a controlled and targeted manner, enhancing their uptake by plants and reducing environmental impact [14]. Furthermore, NPs can be designed to interact with plant cells at a molecular level, promoting plant growth and increasing resistance to stressors [15].

Beyond nutrition, nanotechnology has shown promise in such areas as disease and pest management, plant protection, and seed treatment [16]. NPs can be utilized to enhance the efficacy of pesticides and fungicides, reduce the need for chemical applications, and mitigate the development of resistance in pests and pathogens [17]. Additionally, the use of nanomaterials for controlled-release systems in agrochemicals can minimize the environmental footprint of these substances by ensuring their gradual release and reducing the frequency of application [18].

Nanoparticles can be obtained from a variety of materials, such as metals, carbon, polymers, and ceramics, with each offering distinct properties that can be tailored for specific agricultural applications [19]. For instance, studies have demonstrated that metal nanoparticles, such as those made from zinc, copper, and titanium, can improve a plant’s antioxidant capacity, thus enhancing its ability to withstand oxidative damage caused by environmental stressors, can enhance plant stress tolerance through nutrient uptake, can influence hormone regulation, etc. Plants, like wheat, tomato, rice, lettuce, or cucumber, can be growth with an increased yield, thus enhancing productivity and sustainability of crops, in the context of increasing global food demands and climate change [20,21,22,23,24].

This review aims to provide an in-depth examination of the role of nanoparticles as plant biostimulants, focusing on their mechanisms of action, types of nanoparticles, synthesis methods, and their applications in agriculture. The review will explore the current state of research on metal-based nanoparticles in the context of plant growth promotion, stress tolerance, and nutrient management. Additionally, we will investigate the ecological and environmental benefits of using metal-based nanoparticles in agriculture, with a particular emphasis on the role of phytosynthesized nanoparticles as a sustainable alternative to chemically synthesized counterparts. In summary, this review seeks to provide a brief overview of the role of nanoparticles in modern agriculture, emphasizing their potential as biostimulants to enhance plant productivity, resilience, and sustainability in the face of growing global challenges.

## 2. Nanoparticles and Their Properties

The application of nanoparticles in agriculture represents an emerging and transformative field that is rapidly gaining attention due to the potential of NPs to improve plant growth, enhance stress resilience, and increase crop productivity. As the demand for sustainable agricultural practices grows in response to challenges, such as climate change, soil degradation, and population expansion, nanotechnology provides an innovative solution to enhance crop production while minimizing the ecological impact of traditional farming practices [25].

### 2.1. Types of Metal-Based Nanoparticles Used in Agriculture

Nanoparticles composed of metals have gained significant attention in agriculture due to their unique properties and functionality. Metal-based nanoparticles (MBNPs) are composed of various metals, such as zinc (Zn), copper (Cu), iron (Fe), silver (Ag), and titanium (Ti), among others. These metals are known for their essential roles in plant growth and development, and when reduced to the nanoscale, they exhibit enhanced reactivity and a much larger surface area compared to their bulk counterparts, making them highly effective as plant biostimulants [26].

#### 2.1.1. Zinc Oxide Nanoparticles (ZnO NPs)

Zinc is an essential micronutrient for plants, involved in numerous biochemical processes, including protein synthesis, enzyme activity, and chlorophyll formation. Zinc deficiency in plants can lead to reduced growth, poor root development, and low crop yields [27]. Zinc oxide nanoparticles (ZnO NPs) have been widely studied for their ability to enhance plant growth and productivity. ZnO NPs not only provide plants with a readily available form of zinc but also stimulate various physiological and biochemical processes in plants [28]. Applying different dosages of ZnO-based fertilizer on the rice crops resulted in an increasing of the rice yield, due to the dry matter accumulation (10.28–16.45% and 4.21–9.41% at the jointing, 11.05–23.60% and 2.63–6.32% at the heading, and 9.36–12.12% and 3.44–7.00%). The enhanced yield can be explained through the adsorption capacity of ZnONPs, which promotes the uptake of elemental nutrients through its interaction with different ions.

One of the primary mechanisms by which ZnO NPs enhance plant growth is through their ability to improve nutrient uptake. The small size and high surface area of ZnO nanoparticles allow them to penetrate plant tissues more efficiently than bulk zinc, promoting better absorption by roots. Additionally, ZnO NPs can stimulate antioxidant systems in plants, mitigating oxidative stress caused by environmental factors, such as drought, high temperatures, and soil salinity. They can also improve seed germination, root elongation, and plant vigor, leading to overall enhanced crop performance [29]. In our opinion, the beneficial effects are due to the NPs properties (dimension and morphology). They are also correlated with the possibility of reducing abiotic and biotic stress of plants in order to improve membrane integrity, scavenging reactive oxygen species generated by stress, regulating cell division, nutrients transport, and modulating levels of phytochemicals and osmoregulators. Another parameter which must be considered and is in a direct correlation with the effect is application mode, namely root or foliar application. The uptake of these nanoparticles can be directly in the soil, releasing ions that can be taken up by plants, or on to the leaf surface, where they can be absorbed. Furthermore, ZnO NPs have been shown to enhance plant resistance to various environmental stressors, such as heavy metals and UV radiation. By modulating the activity of enzymes that act as antioxidants, ZnO nanoparticles help to protect plants from oxidative damage, improving their ability to tolerate challenging conditions. This characteristic is particularly useful in arid and semi-arid regions, where plants are frequently exposed to environmental stresses [30].

#### 2.1.2. Copper Oxide Nanoparticles (CuO NPs)

Copper is another essential micronutrient for plants, playing an important role in photosynthesis, respiration, and several enzymatic processes. Copper oxide nanoparticles (CuO NPs) are known for their antimicrobial properties, which make them effective in controlling fungal and bacterial pathogens that can harm plants [31]. CuO NPs have been shown to improve plant growth by stimulating nutrient uptake, enhancing photosynthetic activity, and promoting root development. Additionally, they are highly effective in enhancing plant defense systems, particularly against pathogens, pests, and diseases. Depending on the application mode (foliar spray, soil supplementation, hydroponics, in vitro) and treated crops, the effect can be different: for example, the final effect of CuO NPs on different Brassica species is influenced by the concentration and method of application, highlighting the concentration-dependent and method-specific effects; foliar application on *Brassica juncea* L. increased growth, biomass, chlorophyll content, and net photosynthetic rate, while for *B. oleracea* var. capitata, decreased plant weight, water content, and photosynthesis were observed; for the application on *B. oleracea* L. seed as a pre-treatment, the method increased root length [32,33].

The study of Abbasirad and collaborators performed on *Hordeum vulgare* L., Zehak cultivar concluded that the concentration of CuO NPs must be carefully managed, as excessive copper levels can lead to toxicity in plants, causing reduced growth and even plant death [34]. When applied at optimal concentrations (1000 mg/L), CuO NPs can effectively improve plant health by acting as both a nutrient source and an antimicrobial agent, due to the small size and high surface area of NPs, which correlate with a faster release of metallic ions.

Research has also demonstrated that CuO NPs improve the efficiency of nitrogen utilization in plants. Nitrogen is a key element for plant growth, and its efficient use is essential for maximizing crop productivity. CuO nanoparticles enhance the activity of nitrogenase, an enzyme involved in nitrogen fixation, thereby promoting better nitrogen uptake and utilization by plants [35]. This effect can be particularly beneficial in nitrogen-limited soils, where the application of CuO NPs could lead to improved crop yields.

#### 2.1.3. Titanium Dioxide Nanoparticles (TiO_2_ NPs)

Titanium dioxide (TiO_2_) nanoparticles are another class of metal-based nanoparticles with significant potential in agricultural applications. TiO_2_ is widely known for its photocatalytic properties, which allow it to harness light energy to drive chemical reactions. In plants, TiO_2_ nanoparticles have been shown to enhance photosynthesis by improving light absorption and increasing the efficiency of energy conversion [36]. This can lead to increased biomass production, improved growth rates, and higher crop yields [37].

TiO_2_ NPs are also known for their ability to improve plant stress tolerance. Studies performed on grapevine saplings have shown that TiO_2_ nanoparticles (concentration 1 to 100 ppm) can enhance plant resistance to various environmental stressors, such as high salinity, drought, and heavy metal toxicity (when used at the proper concentration—10 ppm; at 100 ppm. phytotoxic effects were observed, particularly under drought stress). This is achieved through the activation of stress-responsive genes and the induction of antioxidant activity [38]. The positive effects of using TiO_2_ NP in different concentrations and shapes can be attributed to their ability to enhance nutrient and water uptake, mitigate oxidative stress, and possibly mimic plant growth hormones, with the effects being directly correlated with the shape of the NPs. Additionally, TiO_2_ NPs can influence root growth and improve nutrient uptake by enhancing the solubility and bioavailability of nutrients in the soil [39].

TiO_2_ nanoparticles are particularly useful in improving the resistance of plants to oxidative stress, which is often triggered by environmental factors, such as UV radiation and extreme temperatures. By enhancing the plant’s antioxidant defense systems, TiO_2_ NPs help reduce cellular damage caused by reactive oxygen species (ROS), improving overall plant health and productivity [15]. Despite the potential advantages, the use of titanium NP pose potential risks, particularly in relation to ROS generation. At high concentrations, the photocatalytic activity of TiO_2_-NPs can result in the overproduction of ROS, causing membrane lipid peroxidation and leading to chlorophyll degradation, resulting in oxidative damage in plant tissues.

The selected application method—whether it involves foliar spraying, seed nanopriming, or soil amendments—greatly affects how these substances are distributed and absorbed by plants. Incorrect application can lead to environmental contamination and unintended exposure of non-target organisms. The overuse of nanofertilizers may result in nutrient runoff, which can contribute to water pollution and the eutrophication of aquatic ecosystems.

#### 2.1.4. Iron Oxide Nanoparticles (Fe_X_O_Y_ NPs)

Iron is an essential element for plant growth, involved in various processes, such as photosynthesis, respiration, and chlorophyll synthesis [40]. Iron oxide nanoparticles (Fe_X_O_Y_ NPs) have shown great promise as plant biostimulants due to their ability to enhance iron availability in soils and facilitate its uptake by plants [41]. Iron deficiency is a common problem in many soils, particularly calcareous and alkaline soils, where iron is often present in insoluble forms that are inaccessible to plants [42]. In addition to their role in nutrient delivery, Fe_X_O_Y_ NPs can stimulate the production of reactive oxygen species (ROS) within plant cells [43]. While ROS are typically associated with oxidative stress, at controlled levels, they can serve as signaling molecules that trigger defense responses in plants. The application of Fe_X_O_Y_ NPs has been shown to enhance plant resistance to biotic stressors, such as pathogens and pests, as well as abiotic stressors, like drought and heat [44].

Fe_X_O_Y_ NPs also enhance the efficiency of photosynthesis by improving the chlorophyll content and the overall health of plant leaves. By promoting more efficient energy capture and conversion, Fe_X_O_Y_ NPs can lead to increased biomass production and higher crop yields, even if they are applied as a foliar, water, or soil treatment [45].

#### 2.1.5. Noble Metal Nanoparticles (Ag NPs, AuNPs)

Silver nanoparticles (Ag NPs) are well known for their strong antimicrobial properties, which make them particularly effective in protecting plants from a wide range of fungal, bacterial, and viral pathogens [46]. In addition to their antimicrobial activity, Ag NPs can enhance plant growth by stimulating antioxidant systems and improving nutrient uptake. The small size of silver nanoparticles allows them to penetrate plant cells easily, where they can interact with cellular structures and promote metabolic processes that enhance growth [47].

Although some debate on their categorization as a “biostimulant” exists, silver nanoparticles have been exhaustively presented by literature data to provide a biostimulant effect when applied in agricultural practices [48,49]. Ag NPs have been used as a natural alternative to chemical pesticides, providing a more sustainable method of controlling plant diseases. Their biocidal properties can help reduce the need for chemical fungicides, which often have negative environmental and health impacts [50]. Additionally, Ag NPs can be used to enhance seed germination, root development, and overall plant growth [41]. However, as with CuO NPs, it is essential to regulate the concentration of Ag NPs to avoid toxicity, as excessive amounts can harm plant tissues [51].

Silver nanoparticles have also been shown to improve plant tolerance to abiotic stressors, such as drought and high salinity. By modulating the expression of stress-related genes and enhancing the production of stress-protective proteins, Ag NPs can help plants withstand harsh environmental conditions and improve crop resilience [52].

While not as commonly encountered as silver nanoparticles, gold nanoparticles (AuNPs) have been shown to enhance seed germination, promote root and shoot elongation, and improve photosynthetic efficiency in several crop species, including wheat, cucumber, and lettuce [53,54,55]. These effects are thought to be mediated by the nanoparticles’ influence on reactive oxygen species (ROS) signaling, hormone regulation, and nutrient uptake. Additionally, AuNPs may contribute to stress tolerance by activating antioxidant defense systems [56].

### 2.2. Characteristics of Nanoparticles Relevant to Plant Interaction

The unique properties of nanoparticles make them highly effective in interacting with plant cells and tissues. Nanoparticles possess several characteristics that influence their behavior in plants, including their size, surface area, charge, and reactivity. Understanding these characteristics is crucial for maximizing the biostimulant effects of nanoparticles and ensuring their safe and effective application in agriculture (Table 1).

One of the most important properties of nanoparticles is their large surface area relative to their volume. Due to their nanoscale size, nanoparticles have an exceptionally high surface area, which allows them to interact more readily with plant cells, tissues, and molecules [57]. The increased surface area of nanoparticles enhances their ability to deliver nutrients, growth regulators, and other bioactive compounds to plants more efficiently than traditional bulk materials. For example, CuONPs with a particle size of 25 nm compared to the nanoparticles containing 50 nm and 250 nm had a notable impact on root biomass, area, length, and volume (mean root dry weight (g/plant)—0.94 to 5.45; mean root length (cm): 18.8 to 38.6); for SeNPs applied to wheat, the absorption of 40 nm NPs was 1.8–2.2 times higher than SeNPs 140 nm and 240 nm; for *Nicotiana xanthi*, AgNPs at 3.5 nm penetrated the cell wall, while 18 nm NPs gathered on the outer surface [58]. The high surface area of nanoparticles also leads to increased reactivity, as the greater number of surface atoms or molecules makes them more chemically active [59]. This reactivity can facilitate interactions with plant cell walls, membranes, and proteins, leading to changes in plant metabolism and growth. Additionally, the increased reactivity of nanoparticles can stimulate plant defense mechanisms, enhance nutrient uptake, and improve resistance to environmental stresses [60].

The size and shape of nanoparticles are critical factors that determine their interaction with plant cells. Nanoparticles in the range of 1–100 nanometers are small enough to penetrate cell walls and move across plant tissues. Smaller particles are typically more efficient at entering plant cells and tissues, making them ideal for delivering nutrients, growth regulators, and other active compounds directly to the plant’s internal structures; this also displays a strong correlation with the application mode (foliar spray, seed nanopriming, soil treatment, etc.) [14].

Spherical nanoparticles, for example, are often more readily absorbed by plant cells due to their uniformity and symmetrical shape, whereas elongated or rod-shaped nanoparticles may have different effects on cellular uptake and plant growth [61]. The shape of nanoparticles can be tailored to optimize their function as plant biostimulants, depending on the specific application. For example, ZnO NPs with crystallite dimensions of a 34.4 round-shape morphology are effective in enhancing plant growth and yield [60].

### 2.3. Synthesis Methods of Metal-Based Nanoparticles

The synthesis of metal-based nanoparticles is crucial for determining their properties and functionality. Various methods are employed to produce these nanoparticles, including chemical, physical, and biological methods. Each method has its advantages and disadvantages, depending on the desired characteristics of the nanoparticles. As the subject is one exhaustively presented in several works [57,62,63,64], we will only briefly present the main routes currently used for nanoparticles synthesis, in order to underline some of the characteristics that could influence their application as plant growth enhancers.

#### 2.3.1. Chemical Synthesis

Chemical synthesis is one of the most commonly used methods for producing metal-based nanoparticles. Techniques, such as sol–gel processes, chemical vapor deposition (CVD), and hydrothermal synthesis, allow for precise control over the size, shape, and composition of nanoparticles [64,65], ensuring the reproducibility of the process. Chemical synthesis is versatile and can be used to produce a wide variety of nanoparticles with specific properties, such as high surface area, stability, and reactivity [66]. However, the use of toxic chemicals and solvents in chemical synthesis may raise environmental and safety concerns.

#### 2.3.2. Physical Synthesis

Physical methods of nanoparticle synthesis involve mechanical processes that break down bulk materials into nanoparticles. Techniques, such as laser ablation, ball milling, and sputtering are commonly used in this category. Physical methods are typically energy-intensive and require specialized equipment but offer high control over particle size and purity [67,68]. These methods are often used when producing nanoparticles from such metals as copper, zinc, and iron.

#### 2.3.3. Biological Synthesis (Green Synthesis)

Biological synthesis, also known as green synthesis, involves using biological systems, such as plants, bacteria, or fungi to produce nanoparticles [69]. This method is environmentally friendly and avoids the use of toxic chemicals, making it a sustainable alternative to traditional synthesis methods. Biological synthesis of nanoparticles can result in nanoparticles with unique surface characteristics that enhance their interaction with plant cells and tissues [70,71,72]. Green synthesis methods have been successfully used to produce a variety of metal-based nanoparticles, including zinc oxide, copper oxide, and silver nanoparticles, which can be used as biostimulants in agriculture [73]. However, biological methods present several bottlenecks in the development of commercially products, one of the most important ones being the ability to implement a reproducible process. Although comparative analyses between the chemically synthesized and green synthesized nanoparticles were not identified in the literature (this being a topic that should be tackled in future studies), the available data regarding the influence of the phytosynthesis process on other applications [73,74,75,76,77] allows the proposal of a superior effect of the phytosynthesized nanoparticles, when compared with other synthesis methods.

## 3. Phytosynthesized Nanoparticles as Plant Biostimulants

Among the various methods of nanoparticle synthesis, phytosynthesis, which uses plant extracts to produce nanoparticles, has gained significant attention due to its eco-friendly, cost-effective, and sustainable nature. This section delves deeply into the role of phytosynthesized nanoparticles (PSNs) as plant biostimulants, exploring their mechanisms, benefits, examples, and the environmental advantages they offer over traditional synthetic methods.

### 3.1. Overview of Phytosynthesis of Nanoparticles

Phytosynthesis refers to the use of plant-derived materials, such as extracts obtained from leaves, roots, stems, fruits, entire plants, and algae, to mediate the synthesis of nanoparticles, by capitalizing on the natural capabilities of plants to reduce metal ions into their metallic state and stabilize them into nanoparticle forms. Unlike traditional chemical or physical synthesis methods, which often require high-energy inputs, toxic chemicals, and environmentally harmful solvents, phytosynthesis offers a cleaner and more sustainable alternative.

**Figure 1:**
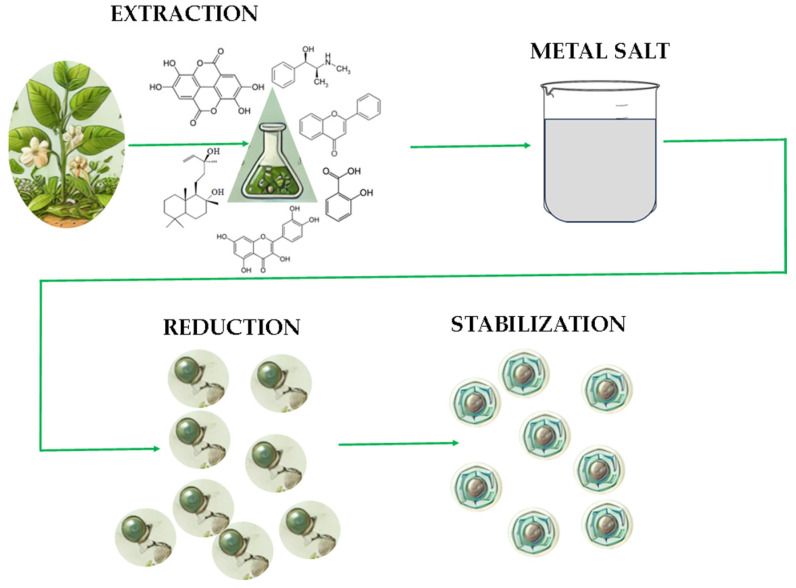
Phytosynthesis process.

In the phytosynthesis process (Figure 1), plant extracts serve as both reducing agents and stabilizers. The secondary metabolites present in plant extracts, such as polyphenols, flavonoids, alkaloids, and terpenoids, play critical roles in reducing metal ions into their corresponding nanoparticle forms, while also stabilizing the nanoparticles and preventing their agglomeration [73]. The plant-derived stabilizers, such as proteins and polysaccharides, help form a protective layer around the nanoparticles, providing steric and electrostatic stabilization. As a result, phytosynthesis not only produces nanoparticles in a more sustainable manner but also enables the design of nanoparticles with specific properties tailored to particular agricultural applications. Despite these obvious advantages, the phytosynthesis process still requires some steps towards a successful standardization of the obtained results. One of the most important steps in this direction is represented by the use of controlled composition extracts, as well as strictly controlled synthesis parameters [73,74,75,76].

The use of plant extracts for nanoparticle synthesis has several advantages over other synthesis methods. Plant extracts are abundant, renewable, and inexpensive, making the process cost-effective. Additionally, the diversity of plant species allows for the synthesis of nanoparticles with a wide range of sizes, shapes, and surface characteristics, making it possible to tune the properties of the nanoparticles for specific agricultural needs. Phytosynthesized nanoparticles have been shown to exhibit superior stability, biological activity, and biocompatibility [71], which makes them particularly attractive for use in agriculture.

### 3.2. Mechanisms of Phytosynthesis

The phytosynthesis of nanoparticles is a complex process involving several stages [75,76]. These stages include the reduction of metal ions, the stabilization of the resulting nanoparticles by the involved phytoconstituents, and their further treatment (e.g., functionalization to enhance their biological activity). Understanding the mechanisms involved in phytosynthesis is crucial for optimizing the synthesis process and ensuring that the nanoparticles exhibit the desired properties for plant biostimulant applications.

The first step in the phytosynthesis of nanoparticles is the reduction of metal ions (such as Ag^+^, Au^3+^, or Cu^2+^) into their respective metallic forms (e.g., Ag, Au, and Cu). This reduction is facilitated by plant-derived compounds, which act as reducing agents. Secondary metabolites in the plant extract, such as polyphenols, flavonoids, and proteins, are capable of donating electrons to the metal ions, reducing them to their zero-valent state [77]. This step is critical for nanoparticle formation and plays a key role in determining the size and shape of the resulting nanoparticles.

Phytochemicals in plant extracts also influence the rate of reduction. For example, smaller phytochemicals tend to promote the formation of smaller nanoparticles, while larger compounds may lead to the formation of larger particles [78]. The concentration of phytoconstituents, the concentration of metal ions in the solution, the temperature, and the pH also play significant roles in determining the size and shape of the nanoparticles formed during the reduction process [77,79,80,81].

Once the metal ions are reduced to their elemental form, the resulting nanoparticles must be stabilized to prevent agglomeration or aggregation. Nanoparticles have a high surface energy, which makes them prone to clumping together, leading to the formation of larger particles. To avoid this, the plant-derived stabilizers in the extract, such as proteins, polysaccharides, and secondary metabolites, coat the surface of the nanoparticles and prevent their aggregation [82]. These stabilizers form a protective layer around the nanoparticles, providing both steric and electrostatic stabilization. The surface of the nanoparticles can also be functionalized with specific chemical groups, such as hydroxyl, carboxyl, or amino groups, which can enhance the interaction between the nanoparticles and plant cells. This functionalization also allows for the controlled release of bioactive substances and improves the bioavailability of the nanoparticles, enhancing their effectiveness as biostimulants [83].

The size and shape of the nanoparticles are critical factors that influence their interaction with plant cells and their effectiveness as biostimulants. The phytochemicals in the plant extract play a key role in controlling the size and shape of the nanoparticles. The size and shape of the nanoparticles also determine their ability to penetrate plant tissues and interact with cellular structures. Smaller nanoparticles tend to have better bioavailability and can more easily penetrate cell membranes, while larger particles may be more effective in promoting surface interactions with plant roots and leaves [61]. The control of particle size and shape is, therefore, an important consideration when designing nanoparticles for agricultural applications. By ensuring reproducible results with the phytosynthesis process, it can achieve a major step in the large application of the process, namely the development of commercially available products.

### 3.3. Benefits of Phytosynthesized Nanoparticles in Agriculture

The use of PSNs in agriculture offers numerous benefits, ranging from improved plant growth and nutrient uptake to enhanced stress tolerance and disease resistance [51,84,85,86]. These benefits make PSNs a valuable tool in modern agricultural practices, where sustainability and eco-friendliness are becoming increasingly important.

PSNs have been shown to promote seed germination, root development, and overall plant growth [84]. The nanoparticles interact with plant cells and tissues, influencing various physiological processes, such as nutrient absorption, photosynthesis, and enzyme activity. For example, silver (Ag) nanoparticles synthesized using *Annona squamosa* extracts have been reported to stimulate the production of chlorophyll, which enhances photosynthesis and accelerates the plant growth of *Phaseolus vulgaris* [87]. Similarly, copper (Cu) nanoparticles (obtained using extract of mangrove leaves, *Avicennia marina*) have been shown to improve the root length of wheat seedlings, which improves the plant’s ability to absorb water and nutrients from the soil. The recorded root length improvement was approx. 73% (compared with the control, at 0.06 mg/mL, and superior to the extract at the same concentration, with a 52.22% improvement) [88]. However, at a higher concentration (0.43 mg/mL), CuNPs significantly inhibited root and shoot lengths. Nanoparticles can enhance the uptake of essential nutrients, such as nitrogen, phosphorus, and micronutrients, by plants. The nanoparticles facilitate the solubilization of nutrients in the soil, making them more bioavailable to plant roots. For example, zinc oxide nanoparticles (ZnO phytosynthesized using *Eucalyptus lanceolatus* leaf extract) have been shown to improve zinc uptake in *Zea mays* L. plants at 200 ppm concentration, which is particularly important in soils with low bioavailable zinc [89]. Nanoparticles also enhance the transport of nutrients across plant cell membranes, improving nutrient absorption efficiency.

In addition to enhancing the uptake of essential nutrients, PSNs can also help plants access previously unavailable nutrients in the soil. By breaking down nutrient complexes and increasing the solubility of minerals, nanoparticles can make nutrients more accessible to plants, thereby promoting growth and increasing productivity [90].

One of the most significant advantages of PSNs is their ability to enhance plant resilience to abiotic stress factors, such as drought, salinity, heavy metal toxicity, and extreme temperatures. Nanoparticles activate various stress-responsive pathways in plants, leading to the production of reactive oxygen species (ROS) and antioxidant enzymes, which help plants cope with oxidative stress [91]. For instance, titanium dioxide (TiO_2_) nanoparticles have been shown to improve drought tolerance by enhancing water retention and modulating gene expression related to stress tolerance [38]. Similarly, copper oxide (CuO) nanoparticles have been reported to increase salt tolerance by improving the plant’s ability to manage osmotic stress [92,93].

### 3.4. Examples of Phytosynthesized Nanoparticles for Plant Growth Promotion

A variety of metal nanoparticles have been synthesized using plant extracts, including silver (Ag), gold (Au), copper (Cu), zinc (Zn), and iron (Fe) nanoparticles.

Silver nanoparticles synthesized from plant extracts, like *Azadirachta indica* (neem) [84], *Picea abies* needles [94], or *Raphanus sativus* L. [95], have demonstrated significant plant growth-promoting properties in studies performed on tomatoes, birch, and grapevine, respectively. These nanoparticles stimulate chlorophyll production, enhance root growth, and improve overall plant health. Additionally, their antimicrobial properties help protect plants from diseases caused by different pathogens.

Gold nanoparticles synthesized using spinach leaf proteins [96] and *Terminalia arjuna* fruit extracts [97] were proven to enhance the seed germination, plant growth, and biochemical parameters of *Spinacia oleracea* L. and *Gloriosa superba*, respectively. Gold nanoparticles also stimulate photosynthetic activity and increase enzyme production, leading to better plant growth and increased productivity [98,99].

Copper nanoparticles phytosynthesized using *Solenostemma argel* leaf extract were proven to mitigate the negative impacts of salt stress and enhance the plant growth-related parameters in a study performed on barley plants [100]. Zinc oxide nanoparticles, developed using *Coriandrum sativum* leaf extract, exhibited a positive effect on the germination rate, plant growth, chlorophyll, and protein content of Bengal gram, Turkish gram, and green gram [101]. Iron oxide nanoparticles, phytosynthesized using *Chenopodium album* and *Fumaria indica* [102], as well as *Psidium guajava* leaf extract [103], were proven to shorten the plant life cycle, and increase chlorophyll content, ascorbate peroxidase, superoxide dismutase, peroxidase, and catalase activities in *Oryza sativa* L. [102], and to increase nutrient availability, shoot length, branch number, shoot diameter, and nitrogen uptake in *Solanum lycopersicum* [103]. Meanwhile TiO_2_ NP obtained from moss biomass decreased the concentrations of stress-related enzymes in saline soil of Chinese spinach (*Amaranthus dubius* L.) [37].

Table 2 presents some examples concerning the application of different types of phytosynthesized nanoparticles with potential biostimulant effects.

### 3.5. Ecological and Environmental Advantages of Phytosynthesized Nanoparticles

The phytosynthesis of nanoparticles offers a range of ecological and environmental benefits over traditional synthetic methods, making these types of nanoparticles a more sustainable and eco-friendly option for agricultural applications.

First of all, phytosynthesis is a green, sustainable method of nanoparticle production that avoids the use of toxic chemicals, solvents, and high-energy processes [104]. This makes the process an environmentally friendly alternative to conventional chemical synthesis methods, reducing the overall ecological footprint of nanoparticle production. Secondly, phytosynthesized nanoparticles are often more biodegradable than chemically synthesized nanoparticles. As they break down in the environment, they release non-toxic byproducts that can be safely absorbed by the soil, reducing the risk of environmental contamination [105]. Furthermore, the use of phytosynthesized nanoparticles in agriculture could reduce the reliance on synthetic fertilizers, pesticides, and herbicides. This will lead to a decrease in chemical residues in crops and the environment, making farming more sustainable and reducing the potential harm to non-target organisms, including beneficial insects and soil microbes [17]. However, further studies are necessary to evaluate the impact of the phytosynthesized nanoparticles on the soil microbiota, as well as to evaluate the selectivity of the developed materials against non-target organisms.

## 4. Mechanisms of Action of Nanoparticles as Biostimulants

The application of nanoparticles in agriculture as biostimulants has gained increasing attention due to their unique characteristics and their ability to improve various plant growth processes. NPs can improve nutrient uptake, stimulate antioxidant defense mechanisms, regulate plant hormones, enhance root development, increase stress tolerance, and influence plant–microbe interactions [90]. By enhancing the physiological and biochemical processes within plants, nanoparticles offer a novel approach to improving plant health, crop yields, and stress resilience [13]. The multifaceted mechanisms through which nanoparticles function as biostimulants needs a thorough presentation, in order to elucidate their effects on plant growth, stress responses, and interactions with soil microorganisms.

### 4.1. Enhanced Nutrient Uptake and Transport

One of the key mechanisms by which nanoparticles exert their biostimulatory effects is by enhancing nutrient uptake and transport in plants [61]. The role of nanoparticles in improving the availability, mobility, and absorption of essential nutrients is well-documented [98]. As plants are highly dependent on an adequate nutrient supply for optimal growth and development, improving nutrient uptake can lead to increased crop productivity and better overall plant health [106].

Nanoparticles significantly increase the solubility of essential nutrients in the soil, which in turn enhances their bioavailability to plants. Nutrients that are typically found in insoluble forms or are poorly available in soil, such as micronutrients, like zinc, copper, and iron, can be rendered more bioavailable in their nanoparticle form. Metal oxide nanoparticles, such as zinc oxide (ZnO), copper oxide (CuO), and iron oxide (Fe_2_O_3_), are particularly efficient in this regard. For instance, ZnO nanoparticles release zinc ions into the rhizosphere, making zinc more readily available to plants, such as tomato, rice, soybean, etc. [29]. This is particularly beneficial in soils with low zinc levels, a condition that often leads to stunted growth and poor crop yield in many regions around the world.

The high surface area of nanoparticles plays a significant role in this process. The large surface area to volume ratio of nanoparticles allows for greater interaction with soil particles, increasing the chances of nutrient release and their subsequent absorption by plant roots [60]. Furthermore, nanoparticles can improve nutrient solubility by forming complexes with nutrients, which enhances their dispersion in the soil solution and makes them more readily available to plant roots [107].

Nanoparticles can also enhance the efficiency of nutrient transport into plant cells by activating or facilitating the function of membrane transporters. These transporters, which are proteins embedded in the cell membranes of root cells, are responsible for the uptake of various nutrients and ions from the soil [61]. Metal oxide nanoparticles have been shown to influence the expression of these transporters, effectively boosting the rate of nutrient uptake [108]. For example, zinc nanoparticles have been found to upregulate the expression of zinc transporters in plant roots, improving the uptake of zinc, other essential nutrients, and amino acids by 22.1%, 11.8%, and 77.5%, respectively; they also increased leaf nutrient levels (Zn, Mn, Cu, Fe, and Mg) by between 15.8 and 416.9%, the chlorophyll content by between 22.2 and 24.8%, rubisco enzyme activity by 21.2%, and antioxidant activity by 26.7 to 31.2% [98,109].

Nanoparticles may also enhance nutrient transport by promoting endocytosis, a process through which plant cells engulf and internalize nanoparticles and their associated nutrients [60]. This process is particularly relevant for nutrients that are bound to nanoparticles or need assistance in crossing the root cell membranes.

In addition to enhancing nutrient uptake directly, nanoparticles can also influence root exudation—compounds released by roots into the surrounding soil [110]. These exudates can act to mobilize nutrients, dissolve mineral compounds, and enhance the nutrient availability in the rhizosphere. Nanoparticles can modulate the composition and quantity of root exudates, which improves nutrient dynamics in the soil and facilitates nutrient uptake [90]. For example, metal oxide nanoparticles (such as CeO_2_ (particle dimension 41.7 nm, concentration 100 mg/L)) have been shown to decrease Cd in shoots and to increase total organic carbon in the growth media [111].

By promoting nutrient mobilization, nanoparticles help ensure that plants have access to a larger pool of available nutrients, even in nutrient-deficient or hostile soil environments.

### 4.2. Induction of Antioxidant Defense Systems

Antioxidant defense mechanisms are critical in plants’ ability to cope with the oxidative stress caused by environmental factors, such as pollution, drought, temperature extremes, and pathogen attack. Nanoparticles, particularly metal-based nanoparticles, play a significant role in enhancing antioxidant systems in plants, helping them mitigate the damaging effects of ROS [112].

Many nanoparticles, especially metal nanoparticles, such as Ag, Zn, Cu, and TiO_2_ can trigger controlled ROS production in plant cells, which, in turn, activates antioxidant defense mechanisms [113]. ROS are highly reactive molecules that can damage plant cell membranes, proteins, lipids, and DNA. Under stress conditions, plants produce ROS in excess, but their excessive accumulation can lead to cellular damage and hinder plant growth [114].

Interestingly, nanoparticles have the ability to induce ROS production in a controlled manner, which stimulates the plant’s antioxidant defense mechanisms. This action triggers the upregulation of antioxidant enzymes, such as superoxide dismutase (SOD), catalase (CAT), and peroxidase (POD) [115]. These enzymes neutralize the ROS and prevent cellular damage [116]. For example, silver nanoparticles (AgNPs) have been shown to induce ROS production in plant tissues, which results in an enhanced antioxidant response that improves the plant’s resistance to various stressors [117]. Thorough discussions on the mechanisms of ROS induction by nanoparticles represents the subject of several recently published works [118,119,120], as such we will not further elaborate on these aspects, but will provide some examples on the effects of NPs application. Nanoparticles also promote the activity of antioxidant enzymes, helping plants resist oxidative stress more effectively [15,121]. These enzymes, including SOD, CAT, and POD, play crucial roles in the detoxification of ROS. Studies have shown that the application of nanoparticles can interfere with these antioxidant enzymes [122]. For instance, in *Triticum aestivum* L. plants treated with copper oxide (CuO) nanoparticles phytosynthesized using *Azadirachta indica* leaf extract, an increase in SOD, POD, and CAT activity has been reported, leading to enhanced tolerance to heavy metal (cadmium) induced oxidative stress [123].

The modulation of antioxidant enzymes by nanoparticles can significantly improve plant resilience, not only under normal conditions but also during periods of environmental stress. By enhancing the plant’s ability to scavenge ROS, nanoparticles help maintain cellular integrity and function, which ultimately supports overall plant growth and development.

### 4.3. Modulation of Plant Hormones and Growth Regulators

Plant hormones are essential for regulating various aspects of plant growth, including seed germination, root and shoot development, and responses to environmental stimuli [124]. Nanoparticles influence the production, transport, and signaling of plant hormones, thereby modulating key growth processes and improving plant development.

Auxins and gibberellins are two of the most important plant hormones for promoting growth [125]. Nanoparticles, particularly metal-based nanoparticles, such as iron oxide, copper, nickel, or zinc, but also non-essential elements nanoparticles (such as Ti or Ag), can regulate these hormones [126]. Auxins are critical for promoting root elongation, cell division, and growth. Studies have shown that nanoparticles, like ZnO (applied by foliar spraying) and CuO (as a seed treatment), can increase auxin production, leading to improved root growth and overall plant development, in studies performed on tea plants [127] and on the model plant *Arabidopsis thaliana* [128].

Similarly, nanoparticles influence the biosynthesis of gibberellins, hormones that regulate cell elongation and seed germination. For example, foliar application of ZnO nanoparticles has been shown to enhance gibberellin production, thereby promoting plant growth, seedling vigor, and shoot elongation in sunflower studies [129].

Abscisic acid (ABA) is a plant hormone that plays a central role in plant stress responses, particularly under conditions of water scarcity and salinity [130]. Nanoparticles, particularly those with oxidative properties, can influence ABA biosynthesis, leading to enhanced stress tolerance [131]. For example, silver nanoparticles, applied by seed soaking and plant spraying, have been found to modulate the phytohormone synthesis—ABA (34%), indole-3-acetic acid (IAA, 55%), and gibberellic acid (82%) increased proline production (70%), improving drought tolerance and reducing the detrimental effects of water stress in maize grown under municipal wastewater irrigation [132].

Additionally, nanoparticles were recently reviewed as to their effect on the levels of other stress-related hormones, such as salicylic acid (SA) and jasmonic acid (JA), which are involved in plant defense mechanisms [22]. By modulating these hormone levels, nanoparticles help improve plant resistance to both abiotic and biotic stress.

### 4.4. Promotion of Root Development and Soil Interaction

Root development is essential for plant establishment and growth, as the root system anchors the plant and facilitates the uptake of water and nutrients. Nanoparticles play a significant role in enhancing root growth by influencing cellular processes, like cell division, elongation, and differentiation [61].

Nanoparticles, particularly metal oxide nanoparticles, like zinc oxide and copper oxide, have been shown to promote root elongation [133]. This effect is partly attributed to the regulation of plant growth hormones, such as auxins and gibberellins, which are involved in root development. Nanoparticles can enhance the activity of these hormones, as previously discussed, leading to increased root elongation and overall root mass. The improvement in root growth helps plants access more water and nutrients from the soil, contributing to better plant health and productivity.

Nanoparticles can also influence the soil environment, thus promoting nutrient availability and improving plant–soil interactions. For example, nanoparticles can enhance soil aggregation [134], which improves water retention and nutrient availability in the rhizosphere. Furthermore, nanoparticles can interact with soil microorganisms, fostering beneficial plant–microbe interactions that enhance nutrient cycling and improve soil fertility [90]. This creates a positive feedback loop, where better soil quality leads to better root development, which, in turn, supports plant growth.

Nanoparticles have the potential to significantly enhance plant tolerance to a wide range of abiotic stresses, including drought, salinity, and heavy metal toxicity. These stresses can lead to reduced plant growth, decreased yields, and poor crop quality, making stress tolerance an important trait for improving agricultural productivity [135].

Drought is one of the most critical challenges faced by crops worldwide. Nanoparticles, such as ZnO, have been shown to improve drought tolerance by enhancing the plant’s water retention capacity and promoting the synthesis of osmotic regulators, such as proline and soluble sugars [136,137]. These compounds help plants maintain turgor pressure under water deficit conditions. By improving the plant’s ability to adapt to drought, nanoparticles can significantly enhance crop productivity in arid and semi-arid regions. For example, the application of ZnO nanoparticles on wheat led to an increase in the shoot and root length of nanoparticle-treated seedlings by 4.56% and 29.6%, respectively, compared to non-ZnO-treated seedlings. At the same time, ZnO nanopriming increased the shoot fresh weight, root fresh weight, shoot dry weight, and root dry weight by 42%, 29%, 23%, and 61% in non-stress conditions, respectively, while under drought stress, the same parameters increased by 46%, 24%, 31%, and 20%, respectively, compared with the control treatments [136].

Salinity is another major abiotic stress that limits crop growth. Nanoparticles can mitigate the effects of salinity by improving the plant’s ability to manage ionic stress, enhance ion transport, and regulate osmotic balance. CuO or ZnO nanoparticles, for instance, have been shown to improve salt tolerance by enhancing different properties: foliar application of copper oxide (CuO) and zinc oxide (ZnO) nanoparticles led to a marked increase in leaf area in plants subjected to salt stress. CuO nanoparticles exhibited a more pronounced effect than ZnO, regardless of the salinity level (150 mM or 300 mM NaCl). Specifically, plants treated with CuO NPs displayed an enhancement in leaf area by 38% under 150 mM NaCl and by 97% under 300 mM NaCl, relative to plants exposed solely to salt stress. In contrast, ZnO NPs primarily influenced leaf expansion at the higher salinity level, where a 79% increase in leaf area was observed compared to plants treated only with 300 mM NaCl. In addition to promoting leaf growth, foliar treatment with these metal oxide nanoparticles significantly reduced proline accumulation in salt-stressed plants across both tested salinity levels. CuO NPs reduced the proline content by 78% under 150 mM NaCl and by 54% under 300 mM NaCl, compared to corresponding salt-only treatments. ZnO NPs, on the other hand, demonstrated a comparable reduction (54%) in proline accumulation, but only under the 300 mM NaCl condition. A notable rise in antioxidant enzyme activities was recorded under high salinity (300 mM NaCl) conditions, with increases of 72% for superoxide dismutase (SOD), 44% for ascorbate peroxidase (APX), 56% for catalase (CAT), and a substantial 178% for guaiacol peroxidase (GOPX), relative to non-stressed control plants. Differential responses in enzymatic activity were evident upon nanoparticle application: CuO NPs enhanced SOD activity by 58% in control plants and by 49% in those under 150 mM NaCl stress. Conversely, a reduction in SOD activity was noted in plants co-treated with ZnO NPs and NaCl. Regarding CAT activity, CuO NPs induced a decrease under moderate salt stress (150 mM NaCl), whereas at 300 mM NaCl, they enhanced CAT activity by 39% compared to plants receiving only the salt treatment [138]. Additionally, nanoparticles can reduce the uptake of toxic sodium ions, allowing plants to maintain a balanced ion concentration within their tissues [138].

Heavy metal toxicity is a growing concern in agricultural soils, and nanoparticles offer a promising solution to mitigate this problem. Nanoparticles can reduce the bioavailability of toxic metals, such as cadmium, lead, and arsenic, in the soil, thereby preventing their uptake by plant roots. For example, at the highest foliar application of ZnO NPs (100 mg L^−1^) on wheat, dry weights of shoots, roots, spikes, and grains were increased by 72, 59, 90, and 97% over the control, respectively; for wheat seeds soaked in ZnO NPs (25, 50, 75, and 100 mg/L) compared with the control group, the grain yield of wheat increased, the content of Cd in the grain decreased, the content of chlorophyll a and b increased, and the electrolyte leakage rate decreased [139]. This not only protects plants from metal toxicity but also improves their overall growth under polluted soil conditions.

### 4.5. Interaction with Soil Microorganisms and Plant–Microbe Symbiosis

Nanoparticles can influence the composition and activity of soil microorganisms, which play essential roles in nutrient cycling and plant health. The interaction between nanoparticles and soil microorganisms can enhance beneficial plant–microbe symbioses, such as nitrogen fixation and mycorrhizal associations, leading to improved nutrient availability for plants.

In certain conditions, nanoparticles, especially metal-based ones, have been shown to enhance the growth and activity of nitrogen-fixing bacteria in the rhizosphere [140]. These bacteria, such as *Rhizobium* species, form symbiotic relationships with leguminous plants and convert atmospheric nitrogen into a form that plants can use for growth. Mycorrhizal fungi form mutualistic relationships with plant roots, assisting with water and nutrient uptake, especially phosphorus [141]. Nanoparticles have been shown to stimulate the growth and colonization of mycorrhizal fungi, which improve root development and increase the efficiency of nutrient uptake. For example, the maximum concentrations of N (29.9 g kg^−1^), P (2.20 g kg^−1^), and K (29.73 g kg^−1^) were obtained in the normal irrigation regime for soil fertilized with TiO_2_ NP/Arbuscular Mycorrhizal fungi [142,143,144]. This, in turn, supports overall plant health and growth.

**Figure 2:**
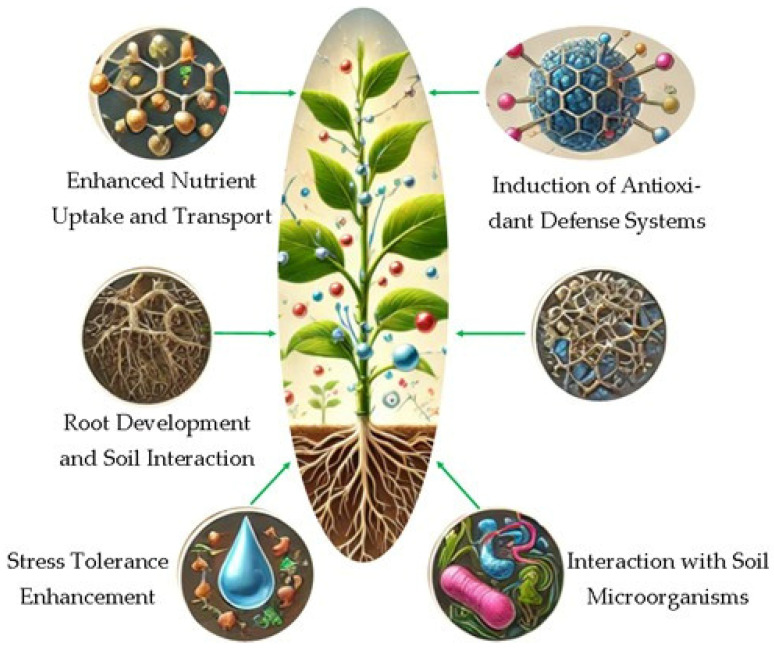
Positive effects of nanoparticles application as biostimulants—main mechanisms.

As can be seen, the use of nanoparticles as biostimulants can offer a wide range of benefits for enhancing plant growth and productivity. Their ability to improve nutrient uptake, induce antioxidant defenses, modulate plant hormones, promote root development, and enhance stress tolerance makes them powerful tools in modern agriculture (Figure 2). Furthermore, nanoparticles interact with soil microorganisms to improve nutrient cycling and plant–microbe symbioses, further enhancing plant health.

## 5. Challenges and Limitations of Nanoparticle-Based Biostimulants

Despite the promising applications of nanoparticles as biostimulants in agriculture, several significant challenges and limitations must be addressed before their widespread adoption. While nanoparticles have demonstrated substantial potential to enhance crop growth, improve stress tolerance, and facilitate nutrient uptake, concerns regarding toxicity, environmental impact, regulatory frameworks, and commercialization barriers continue to hinder their large-scale implementation. Understanding these challenges is essential for the responsible development and application of nanotechnology-based solutions in agriculture.

### 5.1. Toxicity and Environmental Impact of Nanoparticles

One of the most pressing concerns regarding the use of nanoparticles in agricultural applications is their potential toxicity to plants, soil microorganisms, and non-target organisms. Although nanoparticles can act as biostimulants at optimal concentrations, excessive or prolonged exposure may lead to toxicity, disrupting plant metabolism and ecological balance. In plants, nanoparticle toxicity can manifest through oxidative stress, genotoxic effects, and nutrient imbalances [34]. Certain metal-based nanoparticles, such as ZnO and CuO, are known to generate reactive oxygen species, leading to oxidative damage at the cellular level [145]. The excessive production of ROS can impair cellular organelles, disrupt enzymatic activities, and ultimately inhibit plant growth [116]. In addition to oxidative stress, nanoparticles, when used at higher concentrations, may induce genotoxic effects, causing DNA damage, mutations, and chromosomal aberrations [146,147], the long-term consequences of which remain largely unknown. Furthermore, nanoparticle-induced nutrient imbalances can lead to deficiencies or toxic accumulations of essential elements, altering normal physiological processes and reducing crop productivity [34].

Beyond plant toxicity, nanoparticles also pose risks to soil microorganisms, which play a crucial role in maintaining soil fertility and supporting plant growth. Beneficial microbes, such as nitrogen-fixing bacteria and mycorrhizal fungi, contribute to nutrient cycling and organic matter decomposition. However, exposure to high concentrations of nanoparticles may disrupt microbial communities, suppress beneficial microorganisms, and interfere with key enzymatic processes [148]. The antimicrobial properties of certain metal-based nanoparticles, particularly silver (AgNPs) and copper (CuO), can have unintended consequences by negatively affecting the diversity and abundance of beneficial soil microbes [149]. Additionally, some nanoparticles inhibit essential soil enzymes, such as urease and phosphatase [150], leading to a reduction in nutrient availability and a decline in overall soil health.

Another major environmental concern is the potential for bioaccumulation and ecotoxicity. The long-term fate of nanoparticles in agricultural environments remains poorly understood, raising concerns about their persistence in soil and water ecosystems. Some nanoparticles can accumulate in plant tissues, eventually entering the food chain and posing risks to human and animal health [151]. Additionally, nanoparticles can leach into groundwater or be transported through surface runoff, potentially contaminating water sources and affecting aquatic ecosystems [151]. Given their small size and high reactivity, nanoparticles may persist in sediments and disrupt aquatic biodiversity, with potential consequences that are not yet fully explored. Understanding the long-term interactions between nanoparticles and the environment is crucial for assessing their sustainability and safety.

### 5.2. Regulatory Framework and Safety Concerns

The regulation of nanoparticle-based biostimulants is an evolving issue, as existing agricultural policies may not fully account for the unique properties and potential risks associated with nanomaterials. Unlike conventional fertilizers and pesticides, nanoparticles exhibit nanoscale-specific behavior, which may not be adequately addressed by current regulatory guidelines. One of the primary challenges is the lack of standardized regulatory frameworks across different countries, leading to inconsistencies in risk assessment, safety testing, and product approval. Many regulatory agencies struggle to define nanoparticles precisely, with variations in definitions making it difficult to establish clear guidelines for their safe use in agriculture. Additionally, existing risk assessment protocols may not adequately capture the long-term environmental and health effects of nanoparticle exposure, necessitating the development of new testing methodologies.

In the European Union, the REACH (Registration, Evaluation, Authorisation, and Restriction of Chemicals) regulation and associated EFSA (European Food Safety Authority) guidelines have begun incorporating nanomaterials, particularly in the context of food additives and packaging [152]. However, their relevance to agricultural nanomaterials, like biostimulants, remains limited and somewhat ambiguous. The U.S. Environmental Protection Agency (EPA), under the Federal Insecticide, Fungicide, and Rodenticide Act (FIFRA), has required the registration of some nanomaterial-based pesticides [153], but there is no parallel regulatory pathway for nanoparticle biostimulants, which are not always classified as either fertilizers or pesticides.

Moreover, existing risk assessment frameworks typically focus on bulk chemicals and may not account for the unique interactions of nanoparticles with biological tissues, ecosystems, or agro-environmental systems. For instance, conventional toxicity tests often overlook particle size, shape, surface reactivity, and functionalization—key parameters that govern nanoparticle behavior and bioavailability. Additionally, current environmental fate models are not equipped to handle nanoscale dynamics, such as agglomeration, dissolution, or translocation through plant tissues and soil matrices. This creates significant uncertainty in evaluating the long-term impacts of nanoparticle use, particularly regarding their persistence in the environment and bioaccumulation in food chains.

There is also a pressing need to develop nanoparticle-specific safety and efficacy testing protocols that reflect realistic agricultural scenarios, including chronic exposure under field conditions, multispecies environmental interactions, and potential synergistic effects with other agrochemicals. In this context, interdisciplinary collaboration between regulatory agencies, scientific institutions, and industry stakeholders is essential to establish scientifically robust, transparent, and enforceable standards.

Beyond the scientific and regulatory challenges, public perception and consumer acceptance significantly influence the trajectory of nanotechnology in agriculture. There is widespread skepticism about the integration of nanomaterials into food production, stemming from fears about unknown health risks, environmental contamination, and ethical concerns related to technology governance. The lack of long-term epidemiological studies and comprehensive exposure assessments has fueled these anxieties. Without clear and accessible information, consumers may associate nanoparticles with genetically modified organisms (GMOs) or synthetic chemicals, leading to resistance against their adoption in sustainable agriculture.

To address these concerns, transparency and proactive communication are vital. Labeling requirements that disclose the presence of nanoparticles in agricultural inputs and food products could help foster consumer trust, provided they are accompanied by educational initiatives that explain the benefits and risks in a balanced, science-based manner. Public engagement strategies should aim to demystify nanotechnology, highlight its potential to reduce chemical inputs and improve crop resilience, and ensure that stakeholders—including farmers, consumers, environmental groups, and policymakers—are part of the decision-making process.

### 5.3. Inconsistent Results Across Different Crop Species

One of the challenges in the application of nanoparticle-based biostimulants is the variability in plant responses across different crop species. While some crops exhibit significant growth enhancement and improved stress tolerance upon nanoparticle treatment, others show little to no response, making it difficult to establish universally effective formulations. The selective uptake and transport of nanoparticles vary among plant species due to differences in root architecture, metabolic pathways, and physiological traits [55]. Some plants may absorb and translocate nanoparticles efficiently, while others may exhibit restricted uptake, leading to inconsistent results in terms of growth promotion and nutrient enhancement.

Moreover, environmental factors, such as soil composition, pH, organic matter content, and climatic conditions, can significantly influence the behavior and efficacy of nanoparticles in agricultural applications. Soil properties affect the bioavailability and mobility of nanoparticles, altering their interactions with plant roots and microbial communities. For instance, in highly alkaline or acidic soils, nanoparticles may undergo chemical transformations that reduce their effectiveness as biostimulants. Similarly, climatic conditions, such as temperature, humidity, and rainfall, can impact nanoparticle stability and reactivity, further complicating their application under field conditions. The lack of predictability in plant responses underscores the need for further research to optimize nanoparticle formulations based on crop-specific and environmental considerations.

### 5.4. Potential Risks to Soil and Water Ecosystems

The long-term impact of nanoparticles on soil and water ecosystems remains a significant concern, as their accumulation and persistence may lead to unintended ecological consequences. Excessive application of nanoparticles in agriculture could result in soil contamination, altering soil structure, water retention properties, and nutrient availability. Changes in soil microbial communities due to nanoparticle exposure may disrupt ecosystem balance, reducing overall soil fertility and productivity. Additionally, nanoparticles can interfere with soil aggregation processes, affecting aeration and water infiltration, which are critical for plant growth and soil health.

The potential for nanoparticles to enter water bodies through leaching, runoff, or irrigation raises concerns about water pollution and aquatic toxicity. Once introduced into aquatic environments, nanoparticles can interact with biological systems in complex ways, posing risks to fish, algae, and other aquatic organisms. Some nanoparticles have been shown to accumulate in sediments, where they may persist for extended periods, altering water quality and ecosystem stability. Addressing these risks requires the development of sustainable nanoparticle formulations that minimize environmental persistence and toxicity while maintaining agricultural benefits.

### 5.5. Limited Commercialization and Scaling Challenges

Despite extensive research on nanoparticle-based biostimulants, their commercialization remains limited due to economic, technical, and regulatory barriers. One of the primary challenges is the high cost of nanoparticle production, which requires specialized equipment, materials, and expertise. Many synthesis methods currently used in laboratory settings are not easily scalable for large-scale agricultural applications, making it difficult to produce cost-effective formulations. Additionally, the stability and shelf life of nanoparticle-based products need further optimization to ensure long-term usability and commercial viability. Socioeconomic barriers, such as high production costs, limited access to advanced technologies, and low technology literacy among smallholder farmers, may hinder the adoption of nanoparticle-based precision agriculture. Future research should prioritize the development of affordable, user-friendly nanotechnologies and explore inclusive implementation strategies, including farmer training programs, public–private partnerships, and subsidized pilot initiatives tailored to the needs of small-scale farming systems.

Regulatory hurdles further complicate the commercialization of nanoparticle-based biostimulants. The lack of clear approval pathways, safety testing requirements, and standardized labeling protocols creates uncertainty for manufacturers and investors. Companies face significant challenges in bringing nanoparticle-based agricultural products to market, as regulatory compliance processes can be time-consuming and costly. Addressing these barriers through targeted research, regulatory harmonization, and industry collaborations will be essential for facilitating the commercialization and widespread adoption of nanoparticle-based biostimulants.

While nanoparticle-based biostimulants offer exciting possibilities for sustainable agriculture, addressing their challenges and limitations is crucial for their successful integration into agricultural practices. Further research is needed to assess their long-term safety, environmental impact, and regulatory framework. By overcoming toxicity concerns, standardizing regulations, optimizing formulations for different crop species, and ensuring environmental sustainability, nanoparticle-based biostimulants have the potential to revolutionize modern agriculture. A multidisciplinary approach involving scientists, policymakers, industry stakeholders, and farmers will be essential to unlock the full potential of nanotechnology in sustainable crop production.

## 6. Future Directions and Research Needs

### 6.1. Improving the Safety Profile of Nanoparticles for Agricultural Use

Ensuring the safety of nanoparticles in agricultural applications is paramount. Research must focus on assessing the long-term effects of nanoparticle exposure on plants, soil microbiota, and the environment. Developing standardized toxicity assessment protocols will be crucial to evaluating potential risks associated with nanoparticle accumulation and persistence in soil and water ecosystems. Additionally, designing biodegradable or environmentally friendly nanoparticles that degrade into harmless byproducts can help mitigate concerns about their long-term impact.

Another important aspect is understanding nanoparticle interactions at the molecular level within plant systems. Researchers should investigate how nanoparticles influence plant metabolic pathways, gene expression, and stress responses to identify safe concentration thresholds. Furthermore, comparative studies between chemically synthesized and phytosynthesized nanoparticles will help determine which types are safer and more sustainable for agricultural use. Future research must also focus on nanoparticle persistence, accumulation in edible plant tissues, and potential impacts on human and animal health when these crops enter the food chain.

Furthermore, interdisciplinary collaboration among toxicologists, agronomists, and environmental scientists is needed to establish regulatory guidelines for safe nanoparticle usage. Public awareness and education campaigns can also play a role in promoting responsible adoption by addressing misconceptions and ensuring transparency in nanoparticle-based agricultural practices. By improving risk assessment methodologies and regulatory oversight, researchers can build consumer confidence and facilitate the safe integration of nanotechnology into farming systems.

### 6.2. Developing Advanced Nanomaterial Synthesis Methods

The development of more efficient and sustainable nanoparticle synthesis methods is crucial for their large-scale application in agriculture. Traditional chemical and physical synthesis methods often require high energy inputs and the use of toxic solvents, raising concerns about environmental sustainability. Green synthesis approaches, particularly phytosynthesis, offer an eco-friendly alternative by utilizing plant extracts as reducing agents for nanoparticle formation. Further research is needed to optimize phytosynthesis protocols, improve nanoparticle stability, and ensure reproducibility across different plant species and environmental conditions. One of the most important aspects that should be addressed is represented by the use of standardized-composition extracts for the process. This, in turn, could be achieved using alternative approaches, such as in vitro plant propagation [154].

Advancements in nanomaterial engineering should also focus on functionalizing nanoparticles to enhance their efficiency in agricultural applications. Surface modifications, doping with beneficial elements, and encapsulation techniques can improve nanoparticle uptake, controlled release properties, and target specificity. Furthermore, the scalability of synthesis methods should be addressed to enable cost-effective commercial production of nanoparticle-based biostimulants and fertilizers. Additional research into hybrid nanomaterials—combining organic and inorganic components—could lead to novel applications, such as self-regulating nanostructures that respond to environmental changes to optimize plant growth.

Further advancements in biogenic synthesis techniques could provide sustainable alternatives to conventional nanoparticle production. By integrating microbial, fungal, and algal sources into nanoparticle synthesis, researchers may discover new pathways for producing functionalized nanomaterials with enhanced biological activity. Exploring renewable feedstocks, such as agricultural waste and biomass, as raw materials for nanoparticle production could also contribute to a circular economy model and further reduce environmental impact.

### 6.3. Nanoparticle–Plant–Microbe Interactions and Synergies

Understanding the interactions between nanoparticles, plants, and soil microbes is crucial for optimizing their beneficial effects while minimizing unintended consequences. Research should focus on how nanoparticles influence rhizosphere microbial communities, as these interactions play a critical role in nutrient cycling, disease suppression, and plant growth promotion. Studies on the synergistic effects of nanoparticles with beneficial microbes, such as nitrogen-fixing bacteria and mycorrhizal fungi, can lead to novel bio-nanotechnological approaches for enhancing soil health and plant productivity.

Moreover, researchers should explore how different nanoparticle formulations affect microbial diversity, enzyme activity, and soil respiration. Addressing these knowledge gaps will help develop nanoparticle-based solutions that support rather than disrupt soil microbial ecosystems. Advanced metagenomic and metabolomic techniques can provide deeper insights into microbial responses to nanoparticle exposure, guiding the design of more microbiome-friendly nanomaterials. Additionally, it should be investigated the potential of nanoparticles to enhance beneficial microbial biofilms on plant roots, which could contribute to better nutrient acquisition and stress tolerance. As is the case for other types of nanoparticles, future research should also focus on potential detrimental side-effects, such as potential soil accumulation and long-term ecotoxicological effects, evaluating their impact on soil microbial communities, plant health, and food safety over time, as well as potential mitigation strategies, if needed.

### 6.4. Integrating Nanoparticles into Precision Agriculture Technologies

The integration of nanoparticles into precision agriculture has the potential to revolutionize modern farming practices. Smart nanosensors embedded in soil or plant tissues can provide real-time monitoring of nutrient levels, moisture content, and plant health, enabling farmers to make data-driven decisions for optimizing resource use. Research in this area should focus on developing cost-effective and durable nanosensors that can be deployed in field conditions. Advanced nanoscale monitoring systems could help identify nutrient deficiencies or pest infestations before visible symptoms appear, allowing for targeted interventions that reduce chemical inputs and enhance crop yields.

Another promising direction is the development of nanoparticle-based delivery systems for controlled-release fertilizers and pesticides. By encapsulating agrochemicals within nanoparticles, it is possible to achieve slow and targeted release, reducing the overall input of chemicals and minimizing environmental contamination. Future studies should explore the potential of nanocarriers for delivering bioactive compounds, such as plant hormones and biopesticides, to enhance crop resilience and productivity. Integrating nanoparticles into seed coatings could improve germination rates, enhance seedling vigor, and provide early protection against soil pathogens and environmental stressors [155].

Additionally, integrating nanoparticles with Internet of Things (IoT) and artificial intelligence (AI) technologies can enhance farm management strategies. AI-driven analysis of nanosensor data can help predict disease outbreaks, optimize irrigation schedules, and improve yield forecasting. Research efforts should focus on developing user-friendly platforms that allow farmers to harness the benefits of nanotechnology with minimal technical expertise. The potential for blockchain technology to track and verify nanoparticle applications in agriculture could also contribute to transparency, traceability, and regulatory compliance.

### 6.5. Future Perspectives on Phytosynthesized Nanoparticles in Sustainable Agriculture

Phytosynthesized nanoparticles offer a promising avenue for sustainable agriculture, given their eco-friendly synthesis process and potential for plant growth enhancement. Future research should aim to expand the range of plant species used for nanoparticle synthesis and assess their effectiveness under different soil and climatic conditions. Identifying plants with high bio-reducing and stabilizing capacities will be essential for optimizing phytosynthesis efficiency. Additionally, researchers should explore how the biochemical composition of different plant extracts influences nanoparticle properties and their interactions with plant cells

The future of nanoparticle-based biostimulants in agriculture depends on addressing key research gaps and overcoming current challenges. By investing in research and development, the agricultural sector can harness nanotechnology to enhance food security, reduce environmental impact, and promote resilient farming systems for future generations.

## 7. Conclusions

The review highlights several significant findings regarding the role of nanoparticles in agriculture. First, the ability of metal-based nanoparticles, particularly those synthesized through green methods, such as phytosynthesis, to enhance plant growth and stress tolerance is well documented. Nanoparticles can improve nutrient uptake efficiency, activate antioxidant defense mechanisms, and modulate phytohormone levels, thereby acting as effective biostimulants. Moreover, the unique physicochemical properties of nanoparticles, including their high surface area, tunable surface charge, and functionalization capabilities, enable them to interact efficiently with plant cells and soil microbiota.

While the current knowledge underscores the promising potential of nanoparticles as biostimulants in crop production, translating these findings into practical, sustainable solutions requires a targeted and strategic research agenda. Rather than merely summarizing existing knowledge, it is critical to identify and prioritize the key areas where focused scientific inquiry can resolve present limitations and catalyze meaningful innovation in agricultural nanotechnology.

First, establishing comprehensive and standardized toxicity and safety assessment protocols remains a top priority. Existing data are fragmented, and long-term studies on nanoparticle persistence, accumulation in edible plant tissues, and ecological impact are lacking. Future research must address these gaps by developing nanomaterial-specific environmental fate models, chronic exposure studies, and validated biomarkers of nanoparticle bioactivity across diverse crop species and soil types.

Second, the standardization and scalability of green synthesis methods—particularly phytosynthesis—must be advanced. While phytosynthesized nanoparticles show promising biocompatibility and environmental advantages, variability in synthesis outcomes due to differences in plant extract composition limits reproducibility. Future efforts should focus on identifying optimal plant sources, refining extraction parameters, and developing quality control standards that enable industrial-scale production without sacrificing efficacy or safety.

Third, mechanistic studies into plant–nanoparticle and nanoparticle–microbiome interactions are essential. It is not yet fully understood how different nanoparticle formulations modulate plant physiology, root exudation, or microbial community structure. Elucidating these mechanisms will help design biostimulants that support not only individual plant health but also overall soil ecosystem function.

Fourth, functional integration with precision agriculture technologies offers an exciting frontier. Research should prioritize the development of smart delivery systems for controlled release, nanosensors for in-field diagnostics, and AI-driven platforms for data interpretation. These technologies will enhance the spatial and temporal precision of biostimulant application, improving resource efficiency and minimizing environmental impacts.

Fifth, economic and techno-economic analyses must be expanded to evaluate the feasibility of nanobiostimulant deployment at scale. Studies comparing the cost-effectiveness of nanoparticle-based solutions versus conventional inputs, under varying agronomic and climatic conditions, are essential to support decision making by farmers, investors, and policymakers.

Finally, regulatory science must be embedded in the research process from the outset. Clear, harmonized definitions and guidelines for nanoparticle classification, testing, and labeling are urgently needed. Researchers should actively collaborate with regulatory bodies to generate data that inform adaptive, evidence-based policy frameworks. Moreover, socio-ethical dimensions, including consumer perception, should be incorporated into product development and public communication strategies.

In conclusion, future progress in the application of nanoparticle-based biostimulants hinges not only on continued innovation but on a coordinated and multidisciplinary approach. By focusing on the above priorities, the scientific community can accelerate the development of safe, effective, and scalable nanotechnologies that align with the principles of sustainable agriculture. The path forward requires deep collaboration among researchers, regulators, industry, and society to ensure that nanotechnology contributes to global food security while preserving ecological integrity.

## Figures and Tables

**Table 1:** Examples of nanoparticles used for enhancing plant growth and protection.

Type of NPs	NPs’ Properties	Type of Crops	Application Mode	Effect	Ref.
ZnO (commercially available)	Spherical, particle size of 20 nm–50 nm; high specific surface area −133.6 m^2^·g^−1^	Brown rice (Japonica variety)	Basal application; dosage of ZnO 3.75 kg·hm^−2^; 7.5 kg·hm^−2^, 15 kg·hm^−2^, 30 kg·hm^−2^, 60 kg·hm^−2^	Increased rice grain yield by 3.24–4.86% and 3.51–5.12%	[28]
ZnO	Particle size of 37 nm	Grains of wheat (*Triticum aestivum* L.) from Giza 168 cultivar	Priming wheat seeds with bulk ZnO or ZnO nanoparticles at a concentration of 60 mg/L	Enhanced the resilience of wheat plants subjected to drought conditions.	[30]
CuO (chemically fabricated by precipitation method)	Particle size between 25.54–25.83 nm	Cucumber (*Cucumis sativus* L.) seeds	Immersion of seeds for 60 min in the solution of 0.30 M and 0.35 M CuO at a concentration level of 100 µg/L before sowing	Significant inhibitory effect on root rot disease, enhancements in the growth and yield characteristics of cucumbers	[31]
CuO (commercially available)	Irregular shape, particle size of 20 and 50 nm; specific surface area—27.67 m^2^·g^−1^	Soybeans (*G. max* (L.) *Merrill*)	Treatment doses of CuO—1 and 10 mg/kg for 21 days	Enhanced soybean development and improved nitrogen assimilation	[35]
Fe_3_O_4_ (commercially available)	Particle size between 80–110 nm; specific surface area ~30 m^2^·g^−1^	Wheat (*Triticum aestivum* L.) from the variety Moscowskaya 35	Treatment of wheat seeds with Fe_3_O_4_ solution for 3 h	Increases the content of Fe, P, and K in leaves, leading to an improvement in plant growth	[45]

**Table 2:** Representative examples of phytosynthesized nanoparticles used for enhancing plant growth and protection.

Type of NPs	NPs’ Properties	Type of Crops	Application Mode	Effect	Ref.
TiO_2_ (obtained from moss biomass—*Leucobryum glaucum* (*Hedw.*) *Ångstr.*	Non-uniform size	Chinese spinach (*Amaranthus dubius* L.)	Foliar application of TiO_2_	Decreased the concentrations of stress-related enzymes in saline soil	[39]
TiO_2_ (grapevine leaf extract)	Synthesis of green TiO_2_: under magnetic stirring, 2 mL of grapevine leaf extract were combined with 50 mL of 4 mM TiCl_4_, at 80 °C for 24 h. Spherical shape with heterogeneous distribution from 16–23 nm	Three rootstock varieties: Kober 5 BB (*V. berlandieri × V. riparia*) 41 B (41 B Millardet Et de Grasset) (*Vitis vinifera* L. *cv. Chasselas × V. berlandieri* 1103 P (1103 Paulsen) (*V.* *berlandieri × V. rupestris*)	Foliar spray application of TiO_2_ at conc. of 0, 1, 10, and 100 ppm, using 25 mL per/plant	Reduced oxidative damage in grapevine saplings through the regulation of antioxidant defense systems	[40]
AgNP from neem (*Azadirachta indica*) leaf extracts	Synthesis of green AgNP: A 1:1 *v*/*v* ratio of tomato extract to AgNO_3_ (1 mM) was used, for 1 h in an incubator at various temperatures. Spherical to oval shape; particle size between 10–30 nm	Seeds from two Tomato varieties: Nadar and Naqeeb	Seeds soaked for 2 h in AgNP solutions of 5, 10, 15, 20, 25, and 50 ppm concentrations	Enhanced the germination rate and growth of tomato plants, leading to increased production of chlorophyll, carotenoids, alkaloids, and flavonoids.	[88]
CuNP from leaf extract of mangrove—*Avicennia marina* (*Forssk.*) *Vierh*	Synthesis of green CuNP: 10 mL of *Avicennia marina* leaf extract mixed with a 100 mL solution of 4 mM of CuSO_4_·5H_2_O for 3 h at 70 °C Nanoparticle size approx. 11 nm	Wheat plant (*Triticum aestivum* L.) from Egyptian Sakha 93 variety	Foliar spray treatment application of CuNPs (at 0.06 and 0.43 mg/mL with a treatment volume of 15 mL) for 4 weeks	Enhancement of root development and increased chlorophyll levels observed with treatment of 0.06 mg/mL CuNP	[90]
AuNP from spinach (*Spinacia oleracea* L.) leaf extract	Uniform particle size distribution and stability in colloidal systems	Spinach seeds (*Spinacia oleracea* L.)	Seeds treated with AuNP solutions with concentrations between 50–300 µM	Enhanced seed germination, plant development, and biochemical metrics at minimal concentrations (max. 200 µM)	[96]
ZnNP from *Coriandrum sativum* leaf extract	Synthesis of green ZnNP: 0.5 mL of *Coriandrum* leaf extract was combined with 50 mL of Zn(CH_3_CO_2_)_2_ under magnetic stirring for 2 h. The dimensions of the crystallites of ZnNP were between 78 and 84 nm; NP size of 100 nm and rod-shaped	Pulse plant: Bengal gram, Turkish gram, and green gram	Used as fertilizer	Demonstrated a beneficial impact on the germination rate, vegetative growth, chlorophyll levels, and protein concentrations	[101]

## Data Availability

Data sharing is not applicable. No new data were created or analyzed in this study.

## Abbreviations

The following abbreviations are used in this manuscript:

NPs	Nanoparticles
MBNPs	Metal-based nanoparticles
ROS	Reactive oxygen species
PSNs	Phytosynthesized nanoparticles
SOD	Superoxide dismutase
CAT	Catalase
POD	Peroxidase
ABA	Abscisic acid
SA	Salicylic acid
JA	Jasmonic acid
